# Pathogenicity of Aseptic *Bursaphelenchus xylophilus*

**DOI:** 10.1371/journal.pone.0038095

**Published:** 2012-05-25

**Authors:** Li-hua Zhu, Jianren Ye, Sapna Negi, Xu-ling Xu, Zhang-li Wang, Jin-yi Ji

**Affiliations:** 1 Institute of Forest Protection, College of Forest Resources and Environment, Nanjing Forestry University, Nanjing, People’s Republic of China; 2 Jiangsu Key Laboratory for Prevention and Management of Invasive Species, Nanjing, People’s Republic of China; Centro de Investigación y de Estudios Avanzados, Mexico

## Abstract

Pine wilt is a disease of pine (*Pinus* spp.) caused by the pine wood nematode (PWN), *Bursaphelenchus xylophilus*. However, the pathogenic mechanism of pine wilt disease (PWD) remains unclear. Although the PWN was thought to be the only pathogenic agent associated with this disease, a potential role for bacterial symbionts in the disease process was recently proposed. Studies have indicated that aseptic PWNs do not cause PWD in aseptic pine trees, while PWNs associated with bacteria cause wilting symptoms. To investigate the pathogenicity of the PWN and its associated bacteria, 3-month-old microcuttings derived from certain clones of *Pinus densiflora* Siebold & Zucc. produced *in vitro* were inoculated under aseptic conditions with aseptic PWNs, non-aseptic PWNs and bacteria isolated from the nematodes. Six-month-old aseptic *P. densiflora* microcuttings and 7-month-old *P. massoniana* seedlings were also inoculated under aseptic conditions with aseptic PWNs and non-aseptic PWNs. The results showed that the aseptic microcuttings and seedlings inoculated with aseptic PWNs or non-aseptic PWNs wilted, while those inoculated with bacterial isolates did not wilt. Nematodes were recovered from wilted microcuttings and seedlings inoculated with aseptic PWNs and non-aseptic PWNs, and the asepsis of nematodes recovered from aseptic PWN-inoculated microcuttings and seedlings was reconfirmed by culturing them in NB liquid medium at 30°C for more than 7 days. Taken together, the results indicate that the asepsis of PWN did not cause the loss of pathogenicity.

## Introduction

Pine wilt is a disease of pine (*Pinus* spp.) caused by the pine wood nematode (PWN), *Bursaphelenchus xylophilus* (Steiner & Buhrer) Nickle. Although the first occurrence of pine wilt disease (PWD) was reported in 1905 in Japan [1], PWN was not identified as the causal agent of the disease until 1971 [2]. PWD has spread to other Asian countries such as China and Korea and to Europe (Portugal) [3], [4], [5], [6], and has become a worldwide threat to pine forests and forest ecosystems with great economic losses. PWD kills 1,000,000 m^3^ of pine trees annually in Japan [7], and had damaged approximately 7,811 ha of pines in Korea by 2005 [8]. The direct economic losses caused by PWD in China were estimated at approximately $300 million with indirect economic losses exceeding $3 billion [9].

Despite the significance of this disease, the pathogenic mechanism of PWD remains to be elucidated. Until recently, PWN was believed to be the only pathogenic agent causing the disease [10], [11], [12], [13], [14]. Because the physiological and histological changes of diseased trees occur before a rapid increase in the population of nematodes, Oku *et al*. proposed that other agents might be involved in the pathological process [15]. The possible association of PWN and toxin-producing bacteria was reported later [16]. More recently, bacteria from various genera have been found to be associated with *B. xylophilus* [17], [18], [19], [20], [21], [22]. Certain inoculations indicated that bacteria carried by the PWN may play an important role in the pathogenicity of the disease. For example, Kawazu and Kaneko reported that the asepsis of the PWN caused it to lose its pathogenicity [23]. Han *et al*. reported that inoculating aseptic black pine seedlings with aseptic PWNs or bacteria alone did not lead to browning or wilting, but inoculation with aseptic PWNs combined with the bacteria isolated from PWN resulted in the onset of severe symptoms [19]. Zhao *et al*. discovered that inoculation with bacteria alone did not lead to the development of disease symptoms, but a combination of axenic PWNs and bacteria led to disease, while seedlings exhibited none or weak symptoms when inoculated with axenic PWNs or axenic PWNs combined with the non-pathogenic bacterium [20]. The results led the authors to propose a new hypothesis that PWD was a complex disease induced by both, PWNs and their associated pathogenic bacteria.

Although the inoculation tests outlined above provide apparently convincing evidence of the role of bacteria, some scientists think that a note of caution needs to be applied, as bacteria alone cannot cause the disease [24]. It has been proposed that bacteria associated with PWNs are chance contaminants. Because bacteria exist both inside and outside of the host tree, they are not pathogenic [14]. Therefore, further research in this area is needed. The objective of the present study was to evaluate the pathogenicity of bacteria-free PWNs on axenic plantlets derived from clonal plants of *Pinus densiflora* and seven-month-old *P. massoniana* aseptic seedlings.

## Materials and Methods

### Pine Wood Nematode (Non-aseptic)

Four isolates of *B. xylophilus* (strongly virulent AMA3c1, AN19 and AA3, and weakly virulent YW4) were used in all experiments. The strain AMA3c1 was an inbred line maintained by Lihua Zhu in Jianren Ye's laboratory for 20 generations of full-sibling mating [25]. It was derived from the wild isolate AMA3. All of the wild isolates were established with 20–30 individuals collected from dead pine trees by Huang Ren-e [26]. The origin of the nematode isolates is listed in **Table 1**. The nematodes were subcultured on the fungus *Botrytis cinerea* in a 25°C incubator for further use.

**Table 1:** Origin of the four isolates of *Bursaphelenchus xylophilus.*

Virulence	Isolate	Collection site	Source	Year collected
Strong	AMA3	Anhui, Anqing	Dead *Pinus thunbergii*	2004
	AN19	Anhui, Maanshan	Dead *P. massoniana*	2004
	AA3	Anhui, Anqing	Dead *P. taiwanensis*	2004
Weak	YW4	Yunnan, Dehong	Unknown	2005

### Acquisition of Bacterium-free Nematodes

Populations of the four isolates were multiplied on *B. cinerea* cultures on potato-dextrose-agar (PDA) medium at 25°C for 7 days, then washed onto a piece of filter paper in a Baermann funnel with sterile water and kept there for 12 h. A volume of 10 mL of nematode-containing liquid was collected from the bottom of the funnel and centrifuged at 2919 g for 5 min. The supernatant was discarded and the nematode-containing precipitate was washed several times and resuspended with sterile water. The nematode suspension was poured onto a sterilized cover slip in a petri dish, incubated at 25°C and allowed to lay eggs for 4–6 h, after which the nematodes were discarded. Eggs adhering to the surface of the cover slip were rinsed several times with sterile water to remove the nematodes, and the cover slip was placed in a petri dish containing 15% H_2_O_2_ for 60 min at 25°C. The cover slip was rinsed 3 times with sterile water and placed at 25°C in the dark on the mycelia of *B. cinerea* grown on the PDA medium. To prevent the eggs from drying out, a piece of sterile wet absorbent cotton was placed on the cover slip. This method was used for a total of 12 populations, 3 from each of the 4 isolates used for the experiment. The propagated nematodes were collected with a Baermann funnel under aseptic conditions and were checked for the presence of bacteria by culturing them in nutrient broth (NB) (3 g/L beef extract, 5 g/L peptone and 5 g/L NaCl, pH 7.2 ) liquid medium in a flask for more than 7 days.

### Isolation and Identification of Bacteria from Pine Wood Nematodes

Bacteria were directly isolated from AMA3c1 that had been incubated on a lawn of *B. cinerea* PDA medium at 25°C for 7 days. After harvesting the pine wood nematodes with 10 mL of sterile distilled water per plate, the suspension was centrifuged at 2919 g for 5 min. The supernatant was discarded and the pellet was washed 3 times with sterile distilled water. One mL of the suspension (ca. 20000 nematodes) was ground with a mortar and pestle, and a 1 mL aliquot was removed and serially diluted with sterile distilled water. One hundred µL of each dilution was spread onto NB agar medium and incubated at 30°C for 3 days. Colonies were selected and then further cultured to establish pure cultures. Two bacterial strains were isolated from AMA3c1, and were designated as AMA3c1-1 and AMA3c1-2.

The isolated bacteria were mainly identified on the basis of 16 S rRNA gene sequencing. Total genomic DNA was extracted as described by Sambrook *et al*. [27]. From the extracted genomic DNA, 16 S rRNA genes were PCR amplified with the bacterial universal primers 27F and 1492R. PCR was performed in the following program: one cycle of 5 min at 94°C, followed by 30 cycles of 30 s at 94°C, 30 s at 56°C, and 1 min at 72°C, and one cycle of 5 min at 72°C [28]. PCR products were purified using the TaKaRa DNA Purification Kit (TaKaRa Biotechnology, Dalian, China) according to the manufacturer’s instructions and cloned into the pMD19-T vector (TaKaRa), transformed into *Escherichia coli* JM109, extracted, amplified and purified according to standard procedures, and then sequenced at Spring Ltd. (Nanjing, China). The 16 S rRNA gene sequence similarity was determined using the EzTaxon server [29]. The 16 S rRNA gene sequences were deposited in the NCBI database. The phylogenetic dendrograms were constructed by the neighbor-joining method using the molecular evolutionary genetics analysis (MEGA) software version 4.0.

### Culture of Aseptic *P. massoniana* Seedlings

Mature seeds of *P. massoniana* were soaked in 0.1% KMnO_4_ solution for 12 h and then washed in tap water for 1 h; floating seeds were then discarded. The seed coats of the remaining seeds were removed with sterilized forceps and intact megagametophytes were surface sterilized with 70% ethanol for 30 s, and then soaked in 0.1% aqueous HgCl_2_ (w/v) for 3 min followed by 3 rinses with sterile water. Megagametophytes were then placed on a PDA plate for accelerated germination at a certain distance from each other. The process of accelerated germination was monitored frequently and uncontaminated germinated megagametophytes were transferred to a 500 mL flask containing 200–250 mL Gresshoff and Doy [30] medium (GD) supplemented with 0.5–1.0% activated charcoal and cultured there for 7 months under controlled conditions (temperature 25±2°C, 16 h of light at 2000 lx).

### Culture of Aseptic Microcuttings of *P. densiflora*

The culture of aseptic microcuttings of *P. densiflora* was performed as described by Zhu *et al*. [31]. Cotyledon-hypocotyl explants obtained from 21–28 days old aseptically grown seedlings of *P. densiflora* were cultured on GD medium containing 4.0 mg/L 6-BA (6-benzylaminopurine) and 0.1 mg/L NAA (α-naphthylacetic acid) for 5 weeks for bud induction. Induced axillary buds were subcultured on GD medium supplemented with 0.5–1.0% activated charcoal for elongation. Elongated shoots (10–20 mm long) were excised and cut into 5–8 mm stem sections and then cultured on GD medium supplemented with 2.0 mg/L 6-BA and 0.2 mg/L NAA for proliferation. After 4 weeks of culture, shoots were transferred to GD medium supplemented with 0.5–1.0% activated charcoal for elongation. The shoots were subcultured into fresh medium at one month intervals. All cultures were maintained under the same conditions used for the culture of *P. massoniana* seedlings. All shoots generated from a single seed were identified with the same clonal number.

### Inoculation

#### Preparation of inoculum

The aseptically cultured nematodes were harvested by Baermann funnel under aseptic conditions and the suspension was adjusted to a concentration of 10000 nematodes/mL. One mL of this suspension was cultured in NB liquid medium in a flask for more than 7 days at 30°C to assess for contamination, and the remainder was maintained at 4°C. After verifying that the nematodes were bacteria-free, the suspension at 4°C was used for the inoculation test. The unsterilized nematodes cultured on *B. cinerea* for 5–7 days were harvested 1 day before inoculation and adjusted to the same concentration as that of the aseptic nematodes.

The bacterial isolates obtained from the nematodes were used to test their pathogenicity. The two bacterial suspensions that had been shake-cultured in a 200-mL flask for 2 days at 28°C were adjusted to a concentration of 2×10^6^ bacteria/mL and then mixed in equal volumes.

#### Inoculation of aseptic microcuttings with AMA3c1 and bacteria

Aseptic microcuttings elongated on GD medium for 3 months were collected under aseptic conditions and the shoot tips were cut away. The treated microcuttings were placed into fresh medium, a piece of aseptic absorbent cotton was placed on the wound, and 0.02 mL of the inoculum suspension liquid containing 200 nematodes or 4×10^4^ bacteria (the mixture of two bacterial suspensions) was added to the cotton. Inoculation with sterile water was used as a control. Each treatment was performed 3 times using a minimum of 10 microcuttings. All cultures were then maintained at 30°C under continuous, cool white fluorescent illumination (2000 lx) with a 16 h photoperiod. The number of wilted seedlings was recorded every 2 days until 20 days after inoculation.

#### Inoculation of aseptic seedlings with AMA3c1

The shoot tips of 7-month-old *P. massoniana* aseptic seedlings (10–15 cm tall) were cut away with sterilized scissors under aseptic conditions, and a piece of aseptic absorbent cotton was placed on the wound. Twenty five µL of the inoculum suspension liquid containing 250 nematodes were added to the cotton. Inoculation with sterile water was used as a control. Each treatment was performed using at least 10 seedlings. The treated cultures were then maintained under the same conditions described above. The number of wilted seedlings was recorded every 2-days until 30 days after inoculation.

#### Inoculation of aseptic microcuttings with AMA3c1, AN19, AA3 and YW4

Aseptic microcuttings elongated on GD medium for 6 months were collected under aseptic conditions, and the shoot tips were cut away. The treated microcuttings were placed into fresh medium, a piece of aseptic absorbent cotton was placed on the wound, and 0.025 mL of the inoculum suspension liquid containing 250 nematodes was added to the cotton. Inoculation with sterile water was used as a control. Each treatment was performed using 10 microcuttings. All cultures were maintained under the same conditions as above. The number of wilted seedlings was recorded every 2-days until 20 days after inoculation.

### Recovery of the Nematode from the Wilted Microcuttings

Twenty or 30 days after inoculation, wilted microcuttings or seedlings were removed from the bottle and cut into 2–4 mm sections with aseptic scissors; nematodes were extracted with a Baermann funnel. After 12 h, nearly 1 mL of nematode-containing liquid at the bottom of the funnel was collected. The nematodes from the microcuttings inoculated with aseptic nematodes was recovered under aseptic conditions. The recovered nematodes were assessed for bacterial contamination by culturing 200 µL of each suspension in NB liquid medium at 30°C for more than 7 days. The number of recovered nematodes was counted under the microscope.

### Statistical Analysis

SPSS 13.0 software (SPSS, Inc., Chicago) was used for variance analysis. All the data were expressed as the mean ± standard deviation.

## Results

### Acquisition of Bacterium-free Nematodes

Nematode eggs were placed on the mycelia of *B. cinerea* after surface sterilization with H_2_O_2_. After approximately 2–3 weeks, the fungal mats disappeared from the plate. The nematodes were extracted and the results of surface sterilization were assessed. Bathing PWN eggs in 15% H_2_O_2_ for 60 min was shown to lead to complete asepsis. A total of 12 populations were assessed in NB liquid medium for more than 7 days, and only one population from AA3 was not bacterium free.

### Identification of Bacteria

The 16S rRNA gene sequence analysis identified strains AMA3c1-1 and AMA3c1-2 as *Pseudomonas* sp. and *Rhizobium* sp., respectively. The 16S rRNA gene sequences of these two bacterial strains were deposited in the NCBI database, and the accession numbers were JQ419489 and JQ419490, respectively.

### Pathogenicity of Aseptic AMA3c1 on Aseptic *P. densiflora* Microcuttings

**Figure 1:**
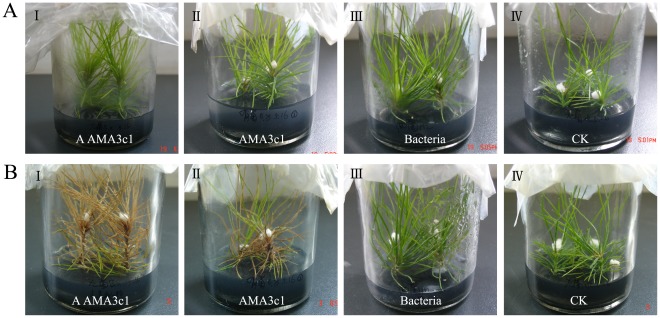
Symptoms of tissue-cultured microcuttings of *Pinus densiflora* 3 days (A) and 18 days (B) after inoculation with the pine wood nematode and bacterial strains isolated from the nematodes: (I) aseptic AMA3c1, (II) unsterilized AMA3c1, (III) bacterial strains, (IV) aseptic water.

The shoot tips of aseptic microcuttings from clones 10-4, 16-2 and 1-A of *P. densiflora* were cut down and inoculated with aseptic AMA3c1, unsterilized AMA3c1 and the mixture of the two strains of bacteria. Microcuttings inoculated with aseptic AMA3c1 and with unsterilized AMA3c1 wilted, whereas those inoculated with bacteria and the control survived for 20 days after inoculation (**Figure 1** **A &** **Figure 1** **B,** **Table 2**). There were no differences in wilting symptoms between the microcuttings treated with aseptic nematodes and those exposed to unsterilized nematodes. In the case of clone 10-4, 4 of 18 microcuttings wilted 6 days after inoculation with aseptic AMA3c1 and another 13 microcuttings wilted during the following 20-day period. In the case of clone 16-2, 6 of 20 microcuttings wilted 6 days after inoculation with aseptic AMA3c1 and another 14 microcuttings wilted 14 days after inoculation. In clone 1-A, the presence of bacteria caused earlier symptoms, but aseptic nematodes eventually wilted 80% of the microcuttings over a 42-day period.

**Table 2:** Wilting ratios of tissue-cultured microcuttings of *Pinus densiflora* inoculated with AMA3c1 and number of nematodes recovered from the microcuttings.

		Wilting rates								Recovery of nematodes/microcutting
Inoculum	Clone	6 d	8 d	10 d	12 d	14 d	16 d	18 d	20 d	20 d
aseptic AMA3c1	10-4	4/18	6/18	10/18	14/18	16/18	17/18	17/18	17/18	442±166 b(240-687)
	16-2	6/20	15/20	18/20	18/20	20/20				1177±765a(6-2106)
	1-A	0	0	2/10	2/10	2/10	2/10	3/10	4/10	N
unsterilized AMA3c1	10-4	3/22	8/22	14/22	17/22	20/22	20/22	20/22	20/22	55±74 c(5-239)
	16-2	5/20	14/20	15/20	17/20	17/20	17/20	17/20	17/20	43±39 c(6-110)
	1-A	0	0	3/10	3/10	3/10	3/10	5/10	7/10	N
bacteria	10-4	0	0	0	0	0	0	0	0	N
	16-2	0	0	0	0	0	0	0	0	N
	1-A	0	0	0	0	0	0	0	0	N
ck	10-4	0	0	0	0	0	0	0	0	N
	16-2	0	0	0	0	0	0	0	0	N
	1-A	0	0	0	0	0	0	0	0	N

1. Data were recorded in the 20 days after inoculation. Values represent the mean + SD. Means in a column followed by different letters are significantly different according to Duncan’s multiple range test (*P*≤0.01).

2. “N” means not done.

### Pathogenicity of Aseptic AMA3c1 on Aseptic *P. massoniana* Seedlings

**Figure 2:**
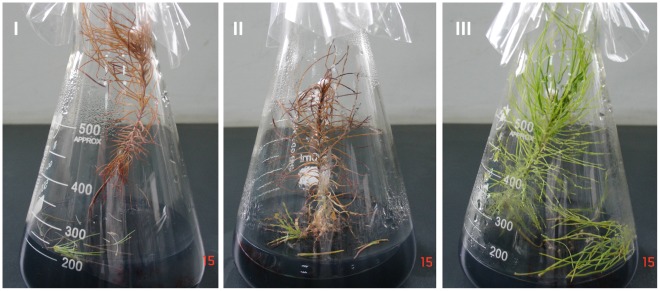
Symptoms of 7-month-old *Pinus massoniana* aseptic seedlings 22 days after inoculation with (I) aseptic AMA3c1, (II) unsterilized AMA3c1 and (III) aseptic water.

In 7-month-old aseptic *P. massoniana* seedlings, inoculation with PWNs caused typical pine wilt symptoms, while plants inoculated with sterile water remained healthy (**Figure 2**). Eighteen of the 20 seedlings inoculated with aseptic AMA3c1 wilted within 30 days after inoculation, and the fastest wilt occurred 6 days after inoculation. Eight of 10 seedlings wilted after inoculation with unsterilized AMA3c1 (**Table 3**). These results suggest that asepsis of the inbred strain AMA3c1 did not result in the loss of pathogenicity.

### Pathogenicity of Aseptic AMA3c1, AN19, AA3 and YW4 on Aseptic *P. densiflora* Microcuttings

**Table 3:** Wilting ratios of 7-month-old P. massoniana aseptic seedlings inoculated with aseptic AMA3c1 and unsterilized AMA3c1, and number of nematodes recovered from the seedlings.

Inoculum	Wilting ratio (%)	Recovery of nematodes/seedling
aseptic AMA3c1	90(18/20)	1158±758 a(108–2635)
unsterilized AMA3c1	80(8/10)	85±84 b(13–215)
ck (aseptic water)	0(0/10)	N

1. Data were recorded 1 month after inoculation. Values represent the mean ± SD. Means in a column followed by different letters are significantly different according to T-test (t=−5.017, df=12.747, p<0.001).

2. “N” means not done.

**Figure 3:**
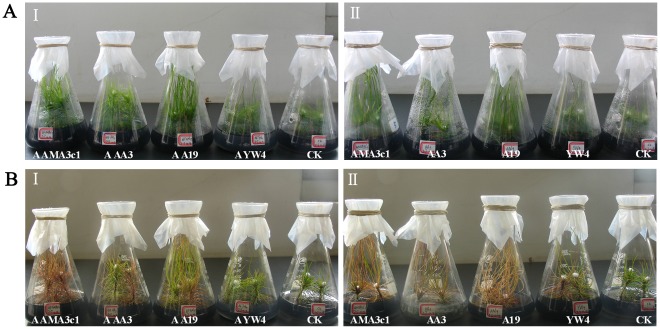
Symptoms of tissue-cultured microcuttings of *Pinus densiflora* 3 days (A) and 18 days (B) after inoculation with 4 isolates of the pine wood nematode: (I) aseptic nematodes, (II) unsterilized nematodes; *AAMA3c1* - aseptic AMA3c1, *AAA3* - aseptic AA3, *AAN19* - aseptic AN19, *AYW4* - aseptic YW4, CK - aseptic water.

Six-month-old aseptic microcuttings from clone 7–7 of *P. densiflora* were inoculated with aseptic AMA3c1, AN19, AA3 and YW4, and unsterilized nematodes. Aseptic PWNs successfully invaded and wilted the microcuttings, as did the unsterilized nematodes 20 days after inoculation (**Figure 3** **A &** **Figure 3** **B**). There was no significant difference in wilting ratios between aseptic and unsterilized nematodes. Of the 4 isolates, AMA3c1 showed the strongest virulence and YW4 showed the weakest virulence against the microcuttings. Inoculation of microcuttings with aseptic AMA3c1, AN19, AA3 and YW4 caused wilting ratios of 90, 70, 60 and 40%, respectively, while these ratios were 80, 70, 60 and 40%, respectively, after inoculation with the corresponding unsterilized nematodes (**Table 4**).

**Table 4:** Wilting ratios of tissue-cultured microcuttings of *Pinus densiflora* inoculated with AMA3c1, AN19, AA3 and YW4, and number of nematodes recovered from the microcuttings.

Inoculum	Wilting ratio (%)	Recovery of nematodes/microcutting
aseptic AMA3c1	90	364±355 ab(110–990)
aseptic AN19	60	229±316 ab(18–846)
aseptic AA3	70	525±455 a(70–1144)
aseptic YW4	40	207±37 ab(165–252)
unsterilized AMA3c1	80	66±52 b(1–135)
unsterilized AN19	70	56±77 b(4–208)
unsterilized AA3	60	69±56 b(10–113)
unsterilized YW4	40	75±77 b(2–182)
ck (aseptic water)	0	N

1. Data were recorded 20 days after inoculation. Values represent the mean ± SD. Means in a column followed by different letters are significantly different according to Duncan’s multiple range test (*P*≤0.01).

2. “N” means not done.

### Recovery of the Nematodes

Nematodes were recovered from wilting microcuttings and seedlings inoculated with aseptic PWNs and non-aseptic PWNs. There were significant differences in the number of nematodes recovered between the two types of inocula. The number of nematodes recovered from wilting microcuttings and seedlings was significantly higher in those inoculated with aseptic PWNs than in those inoculated with unsterilized PWNs (**Tables 2** **,** **3** **&** **4**). In the case of the aseptic AMA3c1 inoculum, the average numbers of recovered nematodes from clones 10-4, 16-2, and 7-7 of tissue-cultured microcuttings of *P. densiflora*, and aseptic *P. massoniana* seedlings were 442 (range: 240-687, N=9), 1177 (range: 6-2106, N=10), 364 (range: 110-990, N=6), and 1158 (range: 108-2635, N=13), respectively. On the contrast, the average numbers of recovered nematodes from clones 10-4, 16-2, 7-7 of tissue-cultured microcuttings of *P. densiflora*, and aseptic *P. massoniana* seedlings inoculated with unsterilized AMA3c1 were 55 (range: 5-239, N=9), 43 (range: 6-110, N=10), 66 (range: 1-135, N=6), and 85 (range: 13-215, N=5), respectively. In microcuttings inoculated with aseptic AN19, AA3 and YW4, the average numbers of recovered nematodes were 229, 525 and 207, respectively, while these numbers were 56, 69 and 75, respectively, after inoculation with the corresponding unsterilized nematodes.

The nematodes recovered from wilted microcuttings and seedlings inoculated with aseptic PWNs were cultured in NB liquid medium at 30°C for more than 7 days and no microorganisms grew on the medium.

## Discussion

PWD is a very complex disease, and the role of bacteria in the pathogenesis of PWD is controversial. Although inoculation experiments performed under sterilized conditions [19], [20], [23] and in the field [20], [32] showed that surface sterilized PWNs lose their pathogenicity, Tamura reported that 3-year-old *P. densiflora* inoculated with aseptic nematodes wilted [33]. Bolla and Jordan also reported aseptic PWNs showed no alterations in their pathogenicity against 45-day-old *P. sylvestris* [34]. Kawazu and Kaneko speculated that the different results of field inoculations might be due to the fact that axenic PWNs regain their pathogenicity after being re-contaminated with bacteria during inoculation [23]. Because bacteria exist everywhere in uncontrolled conditions, *in vitro* inoculation using aseptic nematodes, purified bacteria and hosts grown aseptically was more suitable for the accurate study of plant-pathogen interactions.

Obtaining aseptic nematodes was the first crucial step for the accurate study of the interaction between parasitic nematodes and plants. The successful disinfection of PWNs has been achieved using various chemical disinfecting solutions and different types of sterilizing protocols [19], [34], [35], [36]. Because PWNs carry different bacteria on the surface of their body, it is difficult to obtain axenic PWNs. The present study used a simple and efficient method to obtain bacteria-free PWNs that consist of bathing the PWN eggs during the 4–6 h incubation and allowing them to adhere on the cover slip in 15% H_2_O_2_ for 60 min.

Two bacterial strains were isolated and identified as *Pseudomonas* sp. and *Rhizobium* sp. from AMA3c1. According to the literature, the PWN carries bacteria from different genera and the bacterial communities differ among countries. *Bacillus* is predominant in Japan [37], *Pseudomonas*, *Pantoea*, *Stenotrophomonas* in China [19], [20], [38], *Burkholderia*, *Brevibacterium*, *Ewingella*, *Enterobacter*, *Serratia* in Korea [21], and *Burkholderia*, *Pseudomonas* and other bacteria from Enterobacteriaceae are mainly found in Portugal [22], [39]. Although part of the Chinese PWN population was introduced from Japan [40], *Bacillus* was not reported in China except in the work of Tan and Feng [41]. *Bacillus* was also not found in Portugal [22]. Although *Pseudomonas* has been reported as one of the major phylogenetic groups in Portugal and China, it does not indicate that a *Pseudomonas* species is associated with PWN in Portugal because none of the isolated *Pseudomonas* species was found in all sampling areas and they have not been detected in surface-disinfected nematodes by using molecular techniques [22]. The difference of bacterial composition was likely owing to the discrepancy of geographic areas, environmental factors, pine hosts, and vectors [42]. For example, *P. massoniana* is the dominant host in China, *P. thunbergii* is the dominant host in Japan, and *P. pinaster* is the dominant host in Portugal.

*In vitro* experiments showed that inoculation of pine microcuttings and seedlings with bacteria alone did not cause disease, which was in accordance with previous reports [17], [19], [20]. However, aseptic PWNs successfully infected *in vitro*-grown pine microcuttings and seedlings and caused typical pine wilt symptoms. In our first experiment, inoculation of 3-month-old microcuttings derived from tissue-cultured *P. densiflora* with the aseptic inbred nematode AMA3c1 caused wilting. The results were further confirmed by the inoculation of 7-month-old *P. massoniana* aseptic seedlings with aseptic AMA3c1, and microcuttings of *P. densiflora* with aseptic AN19, AA3 and YW4. These results were contrary to those of Kawazu and Kaneko [17], Han *et al*. and Zhao *et al*. [19], [20], who reported that seedlings inoculated with axenic nematodes did not show wilting symptoms or a few showed only minor symptoms of stem shrinkage without needle browning. One possible reason for these discrepancies may be that the observation period in the studies of Han *et al*. and Zhao *et al*. was too short (only 4 days) for the appearance of wilt symptoms [19], [20]. Tamura reported that most of the aseptic *P. thunbergii* seedlings inoculated with aseptic nematodes collapsed, and the fastest wilt occurred 5 days after inoculation with 1500 nematodes on callus tissues formed on seedling root tips [30]. In the present study, the fastest discoloration of leaves or shrinkage of stems was usually observed 6 days after inoculation.

The nematodes were recovered from wilted microcuttings and seedlings that had been inoculated with either aseptic nematodes or unsterilized nematodes, and the asepsis of nematodes recovered from wilted microcuttings and seedlings inoculated with aseptic PWNs was reconfirmed. Surprisingly, the number of recovered nematodes from wilting microcuttings and seedlings inoculated with aseptic PWNs was significantly higher than that from plants inoculated with unsterilized PWNs. Similar observations have previously been reported by Tamura [30]. When the root callus was inoculated with the combination of aseptic nematodes and bacteria, the number of nematodes decreased rapidly and they did not propagate over a 55-day period. It is likely that bacteria killed callus tissues before the reproduction of nematodes [30]. Despite the successful recovery of nematodes from wilting seedlings in the *in vitro* inoculations reported by Kawazu *et al*., Han *et al*. and Zhao *et al*. [43], [19], [20], these authors did not report on the number of nematodes recovered, and it is therefore not possible to determine whether any differences were present. However, Zhao *et al*. [20] performed field inoculation and reported that the number of nematodes per gram of wood in pine trees inoculated with axenic PWNs was lower than that of trees inoculated with axenic PWNs combined with the pathogenic bacterium or with wild PWNs in the control. The reason for the inhibition of nematode propagation in the present study is not clear, and further investigation is therefore required.

In conclusion, the present study demonstrated that inoculation with axenic PWNs caused wilting of young pine microcuttings and seedlings. However, since there are significant differences between the physiology and pathology of adult pine trees and young seedlings, further research on the role played by nematodes and associated bacteria in disease development should be conducted using axenic PWNs and at least two- or three-year-old pine seedlings grown aseptically.
